# Long Term Sugarcane Crop Residue Retention Offers Limited Potential to Reduce Nitrogen Fertilizer Rates in Australian Wet Tropical Environments

**DOI:** 10.3389/fpls.2016.01017

**Published:** 2016-07-12

**Authors:** Elizabeth A. Meier, Peter J. Thorburn

**Affiliations:** Agriculture & Food Unit, Commonwealth Scientific and Industrial Research Organisation (CSIRO), St. LuciaQLD, Australia

**Keywords:** trash, modeling, soil organic matter, environmental N losses, carbon sequestration

## Abstract

The warming of world climate systems is driving interest in the mitigation of greenhouse gas (GHG) emissions. In the agricultural sector, practices that mitigate GHG emissions include those that (1) reduce emissions [e.g., those that reduce nitrous oxide (N_2_O) emissions by avoiding excess nitrogen (N) fertilizer application], and (2) increase soil organic carbon (SOC) stocks (e.g., by retaining instead of burning crop residues). Sugarcane is a globally important crop that can have substantial inputs of N fertilizer and which produces large amounts of crop residues (‘trash’). Management of N fertilizer and trash affects soil carbon and nitrogen cycling, and hence GHG emissions. Trash has historically been burned at harvest, but increasingly is being retained on the soil surface as a ‘trash blanket’ in many countries. The potential for trash retention to alter N fertilizer requirements and sequester SOC was investigated in this study. The APSIM model was calibrated with data from field and laboratory studies of trash decomposition in the wet tropics of northern Australia. APSIM was then validated against four independent data sets, before simulating location × soil × fertilizer × trash management scenarios. Soil carbon increased in trash blanketed soils relative to SOC in soils with burnt trash. However, further increases in SOC for the study region may be limited because the SOC in trash blanketed soils could be approaching equilibrium; future GHG mitigation efforts in this region should therefore focus on N fertilizer management. Simulated N fertilizer rates were able to be reduced from conventional rates regardless of trash management, because of low yield potential in the wet tropics. For crops subjected to continuous trash blanketing, there was substantial immobilization of N in decomposing trash so conventional N fertilizer rates were required for up to 24 years after trash blanketing commenced. After this period, there was potential to reduce N fertilizer rates for crops when trash was retained (≤20 kg N ha^–1^ per plant or ratoon crop) while maintaining ≥95% of maximum yields. While these savings in N fertilizer use were modest at the field scale, they were potentially important when aggregated at the regional level.

## Introduction

Warming of world climate systems is unequivocal (Intergovernmental Panel on Climate Change [IPCC], 2013), linked to the rapid increase since ∼1950 in atmospheric concentrations of greenhouse gases (GHGs), principally nitrous oxide (N_2_O), carbon dioxide (CO_2_), and methane (CH_4_). Sub stantial and sustained decreases in GHG emissions are therefore advisable to limit future climate change. The ‘Agriculture, Forestry and Other Land Use’ (AFOLU) sector generates almost a quarter of global GHG emissions (Intergovernmental Panel on Climate Change [IPCC], 2013), and so can contribute to global GHG abatement. However, unlike other sectors (e.g., industry, transport), the AFOLU sector has potential to mitigate GHG emissions by removing GHGs from the atmosphere in addition to adopting management practices which reduce the rate of emissions. For agricultural activities in this sector, the main option for removing GHGs from the atmosphere lies in fixing atmospheric CO_2_ during photosynthesis then sequestering part of it in soil organic carbon (SOC) as this biomass decomposes (e.g., by retaining instead of removing crop residues; Sanderman et al., 2010; Department of Agriculture, 2013; Intergovernmental Panel on Climate Change [IPCC], 2013; Thorburn et al., 2013a,b; Smith et al., 2014). Agricultural management practices that reduce emissions of N_2_O include appropriate water and N fertilizer management, particularly with the use of appropriate N fertilizer application rates (Thorburn et al., 2010, 2013b). Emissions of CH_4_ from agricultural activities are predominantly associated with livestock production and rice cropping systems. Abatement options for these systems focus on reducing the emissions intensity of livestock production (e.g., through genetic improvement and manipulation of feed composition), and a combination of water and nitrogen management with straw retention for rice systems.

Sugarcane is a widely grown crop: in 2013 ∼1.9 Gt was produced over >26 Mha globally, with more than half of this (∼15 Mha) located in wet tropical environments (Food and Agriculture Organization [FAO], 2016). In many countries, trash has historically been managed by burning it at harvest (Wood, 1991; Thorburn et al., 2012; de Oliveira et al., 2015). However, this practice is increasingly being abandoned to avoid both management problems such as the rapid decline in stalk sucrose if the harvest of burned crops is delayed (Wood, 1991), and health hazards from smoke pollution (Cancado et al., 2006). Trash blankets contain 7–12 Mg dry matter ha^–1^ and 3–5 Mg carbon ha^–1^, providing some potential to sequester SOC (e.g., Blair et al., 1998; Graham et al., 2002; Thorburn et al., 2012; Page et al., 2013). The N in trash blankets (30–50 kg N ha^–1^; Meier et al., 2006b; Robertson and Thorburn, 2007a) may also be available for subsequent sugarcane crops and so potentially reduce the crop’s requirement for N fertilizer. However, N recommendations for sugarcane in different countries generally do not differentiate between burnt and retained trash management (Espironelo et al., 1996; Meyer et al., 2007; Schroeder et al., 2010), raising the possibility that trash blanketed crops may be over fertilized. Reducing N fertilizer rates would reduce the N surpluses (i.e., the difference between N inputs and the N removed in harvested cane and in burnt residues) and so reduce environmental losses of N (Thorburn and Wilkinson, 2013), including N_2_O emissions (Thorburn et al., 2010).

The wet tropics region in North Queensland, Australia, provides an ideal case study region to evaluate the potential for N in trash to substitute for part of the crop’s N fertilizer requirements. The region was an early adopter of trash blanketing to support mechanized harvesting over 30 years ago (Wood, 1991; Robertson and Thorburn, 2007a), so C and N concentrations in these soils may be closer to equilibrium than elsewhere. The sugar industry uses the largest amount of N fertilizer in this region (Thorburn and Wilkinson, 2013), with high N surpluses and well-understood environmental consequences of these high surpluses (Thorburn et al., 2013a,b; Kroon et al., 2016). A better understanding of the fate of N from ongoing trash retention may therefore result in improved N fertilizer recommendations and reduced environmental losses of N. Such an outcome would be particularly valuable as this region is bounded by World Heritage reef areas that are threatened by off-site movement of N (e.g., De’eath et al., 2012; State of Queensland, 2013).

Decomposing trash blankets initially immobilize soil N because trash has a high carbon (C) to N ratio (70:1 to 120:1; Meier et al., 2006b; Robertson and Thorburn, 2007b). Many years (possibly decades) of trash blanketing may therefore be needed before soil C and N cycling settle to a new equilibrium where N immobilized by decomposing trash is matched by N mineralized from the increased soil organic matter (Basanta et al., 2003; Thorburn et al., 2012). When soil organic matter reaches equilibrium, some N recycled from trash may ‘substitute’ for N fertilizer and allow fertilizer applications to be reduced. Because of the long time frames involved in these processes, models are useful tools to investigate whether the N in trash can be used to replace part of the N fertilizer required by sugarcane crops. The Agricultural Production Systems sIMulator (APSIM; Holzworth et al., 2014) has a well-developed capability for simulating soil N and sugarcane growth dynamics over varying time scales (Thorburn et al., 2005, 2010, 2011; Biggs et al., 2013). It was therefore used to supplement the shorter term field experiments from the wet tropics that were used in this study.

The objective of this research was therefore to: (1) parameterise and validate the APSIM model to simulate sugarcane production, soil mineral N (SMN) and SOC dynamics in wet tropical environments; and (2) to identify the potential for the N in trash blankets to substitute for N fertilizer and increase SOC stocks in these environments.

## Materials and Methods

### Overview

The study was conducted in three stages. Firstly, APSIM was parameterized with site-specific data from two small plot field experiments, and was calibrated to predict crop yield and SMN for these experiments. In the second stage, the calibrated model was fitted with site-specific soil parameters, climate and management from four additional independent trash management experiments and was used to predict yield, SMN and SOC at these additional sites in a validation exercise. Validating the model against independent data, i.e., data that was not used to construct the model, is important to provide additional certainty over modeled outputs (Bellocchi et al., 2010). Lastly, scenarios were simulated with the calibrated model to identify the potential for N inputs from trash blanketing to replace part of the crop’s fertilizer requirement and/or for C applied in trash to increase SOC for the scenarios. Below we describe: the field experiments used for model calibration and validation (see section Field Experiments for Model Calibration and Validation); the configuration, parameterization and evaluation of the calibrated model (see section Model Configuration and Parameterization); and the scenarios analyzed (see section Scenarios).

### Terminology

Sugarcane is a perennial crop sown from vegetative cuttings. In the Australian wet tropics, the first (‘plant’) crop is typically sown between March and June, and harvested around the middle of the following year. A succession of ‘ratoon’ crops shoot from the stumps of each harvested crop and are grown for 12–13 months each. The crop loses vigor after 3–5 ratoons and is plowed out after the final harvest. The combination of plant crop, ratoon crops and fallow period is termed a ‘crop cycle.’ Sugarcane can be planted in the field either shortly after the termination of a crop cycle or after a short (4–6 months) fallow. Crop cycles may therefore differ in a number of ways, including the number of ratoon crops that are grown, the duration of each plant or ratoon crop, and the presence or absence of a fallow period. In this study, these complexities were avoided by presenting ‘per crop’ results that have been averaged over all plant and ratoon crops within the crop cycle.

### Field Experiments for Model Calibration and Validation

#### Calibration Data Sets (Small Plot Experiments)

The Agricultural Production Systems sIMulator was parameterized and calibrated using data from two successive ratoon crops grown between October 2001 and November 2003 in small plot (1 m^2^) field experiments with four replicates (Meier et al., 2006b). The experiments were conducted on two sugarcane farms in the Australian wet tropics, near Babinda (17.34°S, 145.92°E) and Innisfail (17.52°S, 146.03°E). The farms were located on different soil types (a Hydrosol and a Ferrosol; Isbell, 2002), and the soil names were used to identify the sites, i.e., the ‘Hydrosol Site’ (HS) at Babinda and ‘Ferrosol Site’ (FS) at Innisfail. These soil types are common in the Australian wet tropics (Isbell, 2002), and had contrasting properties (e.g., organic C, pH, texture; see Supplementary Material Table 1). Rainfall data was measured onsite at FS but all other weather data was obtained from the SILO data base (Jeffrey et al., 2001) for Babinda and Innisfail. The experiments had three treatments: trash (1) retained on the soil surface, (2) incorporated into the soil, or (3) removed from the plots. Soil (0.0–0.3 m) was sampled from the plots across the row-interrow space at 2–3 month intervals for analysis of total SOC, total N, and SMN. The soil samples were also used to calibrate the rate constants for nitrification used in APSIM (Meier et al., 2006a).

**Table 1:** The trash management, N fertilizer rate, soil type, and climate variables applied in model scenarios.

Factor	Description
Climate	Babinda^1^ (weather station 31004; 17.34°S, 145.92°E) Mulgrave^1^ (weather station 31089; 17.09°S, 145.79°E)
Soil	Hydrosol (HS)^2^ (1.0% C, 0.00–0.15 m) Ferrosol (FS)^2^ (2.3% C, 0.00–0.15 m) High C and N Ferrosol (‘HighCN Ferrosol’; 2.9% C, 0.00–0.15 m) Low C and N Ferrosol (‘LowCN Ferrosol’; 1.7% C, 0.00–0.15 m)
Trash	Trash retained on the soil surface Incorporated by mixing 50% of trash into the 0.00–0.20 m soil layer Burning 70% of trash post-harvest
N Fertilizer	Eight rates from 40 to 320 kg urea-N ha^–1^ in 40 kg N ha^–1^ increments applied to ratoon crops/75% of this rate applied to plant crops, i.e., 30/40 to 240/320.

^1^**Jeffrey et al., 2001*; ^2^*Isbell, 2002**.

The yield of crops in the small plot experiments could not be used for model calibration because they were affected by uneven lodging of sugarcane in the field surrounding the small plots. Individual plot yields were highly variable (data not presented) with the result that treatments were not significantly different (Meier et al., 2006b). The commercial mill yield from fields that included the small plot experiments was therefore used to provide a more representative data set for yield calibration. The commercial yields were obtained each year for a longer period (1990–2003) than the small plot experiment (2002–2003). This ensured that commercial yield could be simulated in response to conditions occurring in the wet tropics such as the influence of intense rainfall and cyclones on soil waterlogging and crop lodging (described in section APSIM-Sugar). Although commercial yields were more representative of yields at the sites than the small plot yields, the management of these crops was subject to various assumptions. Crop planting dates, harvesting dates and whether crop residues were burnt were estimated from additional mill data. The rate of N fertilizer used for the commercial crops was not available from mill data and was assumed to be 130 kg urea-N ha^–1^ following the farmers’ standard practice.

#### Validation Data Sets (Validation Sites)

The model (calibrated with small plot experiment calibration data sets) was validated against data sets from four independent field experiments conducted on farms in the region, termed Validation Sites (e.g., termed VS1 for Validation Site 1, etc.).

Yield and SMN data from VS1-3 was obtained from three field experiments that were each conducted for two ratoon crops (i.e., a total of approximately 2 years’ duration each; Thorburn and Goodson, 2005). Treatments in these experiments consisted of trash (1) retained on the soil surface and (2) incorporated in the soil, and were replicated twice. SMN was measured at 4–6 months intervals in each replicate and treatment, thus providing between 8 and 10 measurement points per site (depending on the duration of the experiment). Crops were harvested commercially and yields obtained from the sugar mill in each treatment for each of the two ratoon crops and two replicates, thus providing a total of four yield measurements for each treatment at each site. Activities undertaken by the farmers to manage the trash, N fertilizer and crop were recorded for the sites. Climate data for the purpose of simulating the sites were obtained from the Babinda weather station (described in 2.1.1), which was located between 1.7 and 7.0 km from VS1-3.

Data for VS4 was obtained from a long-term experiment that had been established to compare the effect of trash blanketing and burnt trash management on sugarcane crop yields and SOC (Wood, 1991; Thorburn et al., 2012). The experiment was planted in 1980, then yield and SOC (0.00–0.25 m depth) were measured at the harvest of each crop for 14 years from 1981. The experiment was unreplicated and thus provided a total of 14 annual measurements of SOC and 12 annual measurements of yields from each of the trash blanketed and burnt trash treatments. As with VS1-3, crops were harvested commercially and yields obtained from the sugar mill, and the farmers’ management recorded for the sites. Daily historical climate data for the purpose of simulating the site was obtained from the SILO weather data (Jeffrey et al., 2001) for the experiment location at –18.48°N, 145.87°E.

Full experimental details are described by Thorburn and Goodson (2005) for VS1-3, and by Wood (1991) and Thorburn et al. (2012) for VS4 (termed the Abergowrie site in those papers). Key soil parameters for VS1-4 are listed in the Supplementary Material Table 1.

### Model Configuration and Parameterization

The APSIM cropping systems model (Holzworth et al., 2014) was configured with modules for soil N (SoilN; Probert et al., 1998), soil water (SoilWat; Probert et al., 1998), trash (Residue; Probert et al., 1998; Thorburn et al., 2001), and sugarcane growth (Sugarcane; Keating et al., 1999). All processes were simulated using a daily time step. The site-specific values of model parameters (given in the Supplementary Material Table 1) were derived as described in the following sections. Default values were used in all other cases.

#### APSIM-SoilN

In the APSIM-SoilN module, soil C and N are subdivided into inert, humic, fresh organic matter, and microbial biomass pools that have constant C:N ratios. The C and N in the inert pool do not decompose, while the maximum potential decomposition rate for C from other pools range in the order of years for the humus pool to days for the microbial pool. The rate at which C flows between the pools is determined by fixed turnover rates for each pool. The amount of C decomposed from each pool is split between the receiving pool and evolved CO_2_ according to efficiency coefficients for each pool. The corresponding flows of N between pools are determined by the C:N ratio of the receiving pool. Any shortfall or excess of N results in mineralization and immobilization of mineral N. The rate of potential nitrification was reduced following Meier et al. (2006a).

The APSIM-SoilN module was configured with seven layers to a depth of 1.5 m (four 0.15 m layers above three 0.3 m layers). Site-specific initial SOC and soil N concentrations used to set up the SoilN module were measured at the field sites (Thorburn and Goodson, 2005; Meier et al., 2006b).

#### APSIM-SoilWat

Soil water in the SoilWat module was configured with seven layers as for the SoilN module. The parameters defining soil water flow and retention in the SoilWat module were determined from measurements of the water characteristic of the soils at all the field sites (Thorburn and Goodson, 2005; Meier et al., 2006b).

#### APSIM-Residue

The decomposition of trash was simulated using an optimal temperature for decomposition (opt_temp) of 30°C and potential decomposition rate (pot_decomp_rate) of 0.06 (mass of residue dry matter) day^–1^ (Thorburn et al., 2001). The proportion of dry matter removed by burning trash was set at 70% (Mitchell et al., 2000). In the APSIM model it is assumed that C and N are removed together when residues are burnt, so 70% of C and N were both removed from the system when trash was burnt.

#### APSIM-Sugar

APSIM-Sugar was configured with variety Q124 available in the standard release of the model. Sugarcane yields are usually reported on a fresh weight basis, so predicted cane yields were calculated from the simulated dry weight and assuming a dry matter fraction of 0.30 (Fageria et al., 2010). The model was calibrated to simulate commercial yield at HS and FS by adjusting crop rooting depth and crop responses to waterlogging and lodging following the approach of Biggs et al. (2013). These modifications were then adopted for all simulations. Thus, the maximum rooting depth was set to 1.5 m for simulations with the Mulgrave climate, consistent with root depth measured in other locations (Fageria et al., 2010). For simulations with the high rainfall Babinda climate, the maximum rooting depth was restricted to 0.9 m because crops were unlikely to be water stressed (Biggs et al., 2013) and may have been restricted by waterlogging (Wood, 1991; Smith et al., 2005; Fageria et al., 2010). For all sites, crop growth was reduced in response to waterlogging by decreasing the potential radiation use efficiency (oxdef_photo) of the crop from 1.0 to 0.2 as the fraction of crop roots exposed to waterlogging (oxdef_photo_rtfr) increased from 0.5 to 1.0. Lodging was simulated on specified dates when it was known to occur, or once per plant or ratoon crop if stalk dry weight exceeded 20 Mg dry weight ha^–1^ and daily rainfall was greater than 20 mm (after Singh et al., 2002; Thorburn et al., 2011; Biggs et al., 2013).

### Scenarios

#### Factors Varied in Model Scenarios

The model (calibrated and validated using the data sets described in the Field Experiments for Model Calibration and Validation section) was used to simulate a range of scenarios to investigate whether the C and N added to soil under continuous trash blanketing increase SOC and/or reduce the N fertilizer required by sugarcane crops in the wet tropics. Four different factors (climate, soil type, trash management, and N fertilizer rate) were combined factorially to cover a range of conditions in which sugarcane crops could be grown in the Australian wet tropics (**Table 1**) and are described below.

##### Climate

**FIGURE 1:**
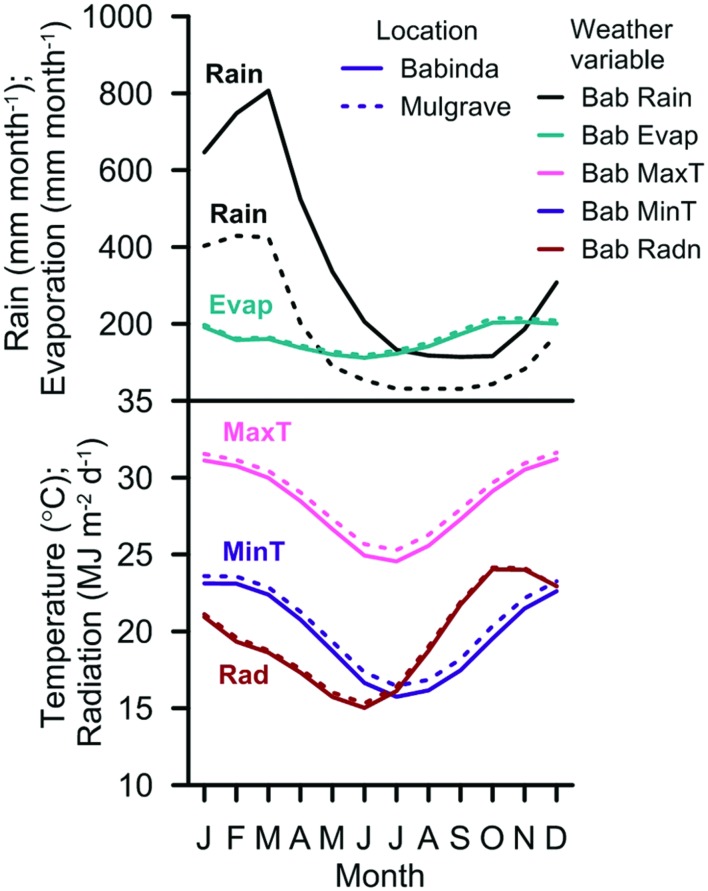
**Average monthly rainfall (Rain), evaporation (Evap), maximum temperature (MaxT), minimum temperature (MinT), and solar radiation (Rad) recorded at the Babinda and Mulgrave weather stations (1906–2005)**.

All model scenarios were based on the historical daily climate records at the Babinda and Mulgrave weather stations for the period 1889–2001 (**Table 1**). Similar climate data were recorded at both weather stations except for rainfall, which was twice as much at Babinda (4,196 mm year^–1^) as for Mulgrave (1,937 mm year^–1^; **Figure 1**).

##### Soil type

Four soils were included in this simulation (**Table 1**), including soils from HS (soil 1) and FS (soil 2), and two additional synthesized variants of the Ferrosol (soils 3 and 4). The variants were created by increasing the SOC of the Ferrosol to 125% in the HighCN Ferrosol scenario, and decreasing organic C to 75% in the LowCN Ferrosol scenario. Other properties of the HighCN and LowCN Ferrosol soils were the same as the Ferrosol soil (given in Supplementary Material Table 1). These two synthesized soils were included because the soil C:N ratio determines the extent of N mineralization, and is important for N management. Variants of the Hydrosol soil were not simulated because crops on this soil were limited by water logging so crops simulated on this soil were less able to respond to simulated differences in soil C and N. The combination of location and soil type were referred to, for example, as the ‘Babinda-Ferrosol’ for the Ferrosol soil subjected to the Babinda weather.

##### Trash management

Three trash management practices were included in the scenarios (**Table 1**); (1) retaining trash on the soil surface (referred to as a trash ‘blanket’), (2) incorporating trash into the soil after harvest, and (3) removing the trash by post-harvest burning. Trash incorporation was included because it may contribute to SOC while minimizing some of the practical problems of trash blankets: e.g., trash being washed off the field in heavy rainfall or floods (Thorburn and Goodson, 2005); waterlogging of poorly drained soils (Wood, 1991); volatilization of NH_4_-N from urea applied to trash blankets (Cantarella et al., 2008). However, there have been few experiments on incorporating trash as a means of avoiding these problems, and these were only conducted for short durations (Ridge et al., 1979; Smith et al., 1984; Thorburn and Goodson, 2005).

##### N fertilizer

Eight N fertilizer strategies were simulated (**Table 1**), in which N fertilizer was applied to ratoon crops at rates ranging from 40 to 320 kg N ha^–1^ in 40 kg N ha^–1^ increments. The amount of N fertilizer applied to plant crops was 75% of the ratoon rate. The plant/ratoon combinations of N fertilizer rates are referred to, for example, as the ‘30/40 rate’ for N applied at the rate of 30 kg N ha^–1^ to plant crops and 40 kg N ha^–1^ to ratoon crops. N fertilizer was placed at a depth of 0.1 m as is common practice to avoid losses by volatilization (Macdonald et al., 2014).

#### Simulation Period for Model Scenarios

Crop cycles were simulated for a 6-year period and consisted of a plant crop sown on May 15 and harvested 15 months later, four ratoon crops of 13 months duration each, and a 4.5-month bare fallow period. All management systems were simulated for 108 years. Each combination of practices was simulated with three different starting years (1889, 1891, and 1893), to avoid potential bias in results that could occur if simulated results for the crop cycle coincided with cyclical patterns in weather. Average results for the three start years are presented. For the first four crop cycles, trash was burnt and N fertilizer was applied at the historical conventional rate (the 120/160 rate; Calcino et al., 2000) to simulate crop management before trash blanketing was adopted. The trash and N fertilizer management systems were then applied in the simulations for a further 14 crop cycles (84 years).

#### Evaluation of Model Performance

The performance of APSIM in simulating yield and SMN for the calibration and validation data sets was evaluated using three measures of agreement between predicted and measured variables: (1) Root Mean Square Error (RMSE), (2) Index of agreement (d; Willmott, 1982), and (3) model efficiency (ME; Nash and Sutcliffe, 1970):

$$R\,M\,S\,E=\frac{\Sigma_{i=1}^{n}\,{\left(P_{i}-M_{i}\right)}^{2}}{n}\,\;\;\;\;\;\;\;\;\;\;\;\;\;\;\;\;\;\;\;\;\;\;\;\;\left(1\right)$$

where *P*_i_ is the predicted variable, *M*_i_ is the measured variable, and *n* is the number of measured values. The RMSE has the same units as the quantity being estimated, and the model fit is better for RMSE values closer to zero.

$$d=1-\frac{\Sigma_{i=1}^{n}\,{\left(P_{i}-M_{i}\right)}^{2}}{\Sigma_{i=1}^{n}\,{\left(\left|P_{i}^{\prime }\right|\;+\;\left|M_{i}^{\prime }\right|\right)}^{2}}\,\;\;\;\;\;\;\;\;\;\;\;\;\;\;\;\;\;\;\;\;\;\;\;\;\left(2\right)$$

where $P^{\prime }=P_{i}-\overset{¯}{M}$ and $M^{\prime }=M_{i}-\overset{¯}{M}$. The index of agreement is unitless and the model fit for *d* improves as the value approaches 1.

$$M\,E=1-\frac{\Sigma_{i=1}^{n}\,{\left(P_{i}-M_{i}\right)}^{2}}{\Sigma_{i=1}^{n}\,{\left(M_{i}-\bar{M}\right)}^{2}}\,\;\;\;\;\;\;\;\;\;\;\;\;\;\;\;\;\;\;\;\;\;\;\;\;\left(3\right)$$

The value of ME is unitless. It ranges from -∞ to 1 (optimal), but values between 0 and 1 are considered to demonstrate an acceptable level of model performance.

## Results

### Model Performance

#### Parameterization

##### Yield

**FIGURE 2:**
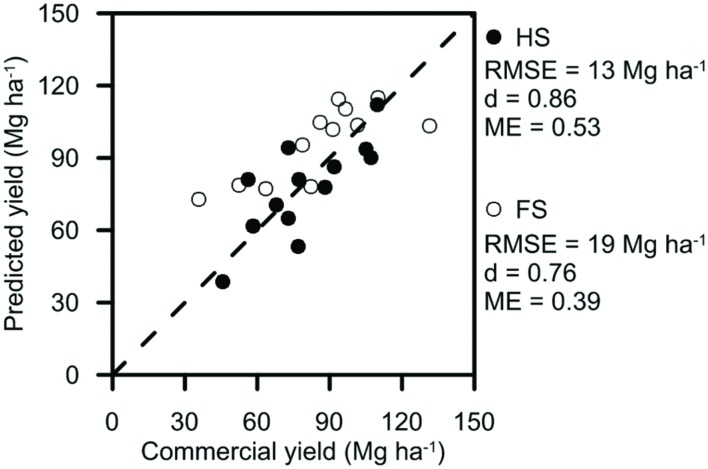
**Measured and simulated commercial yields for each crop during 1990–2003 for the small plot field experiments at the Hydrosol Site (HS) and Ferrosol Site (FS) used to parameterize the model**. Root mean square error (RMSE), index of agreement (d), and model efficiency (ME) are included.

Sugarcane yields ranged from 46 to 110 Mg ha^–1^ at HS and from 36 to 132 Mg ha^–1^ at FS during the period 1990–2003 (**Figure 2**). The crops were subjected to five and seven tropical cyclones during this period at HS and FS, respectively, and the highest commercial yields at each site occurred in years where crops experienced the lowest rainfall (< ∼3,000 mm crop^–1^). After parameterization, the model captured the general response of yield to these conditions (e.g., ME = 0.53 at HS and 0.30 at FS, **Figure 2**). RMSE values for the parameterization were greater than in other studies (e.g., 2 and 5 Mg ha^–1^ in Keating et al., 1999 and Thorburn et al., 2011, respectively), likely because more accurate management information and yield data were available to parameterize simulations of the shorter-term field experiments in those studies than from the mill records for HS and FS.

##### SMN

**FIGURE 3:**
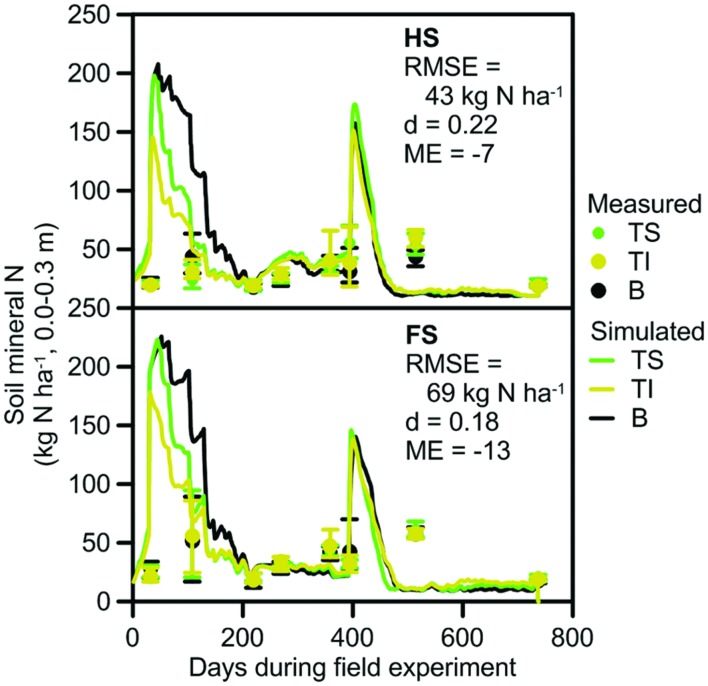
**Soil mineral nitrogen (SMN) for the small plot field experiments at the HS and FS used to parameterize the model**. Measured SMN is shown as symbols with bars for the mean and range of values; simulated SMN is shown as lines. Trash was retained on the soil surface (TS), incorporated (TI) or removed and the soil left bare (B) as a surrogate for burnt trash. RMSE, index of agreement (d) and ME are included.

Average SMN measured in the surface 0.3 m of soil in the small plots ranged from 14 to 70 kg N ha^–1^ at HS and 12 to 95 kg N ha^–1^ at FS (**Figure 3**) during the experiment. There was substantial variability in SMN between replicate plots on individual measurement dates (3–51 kg N ha^–1^ at HS and 3–74 kg N ha^–1^ at FS). The greatest variability in replicates occurred during summer where high rainfall and temperatures may have promoted high rates of mineralization within some plots. An increase in the variability of measured SMN has similarly been observed in other sugarcane experiments during the summer period (Allen et al., 2010) and under wet tropical conditions (Webster et al., 2012). The variability of replicates led to low model performance values (e.g., ME = –7 at HS, **Figure 3**), although the overall trend in SMN was captured.

#### Validation

##### Yield

**FIGURE 4:**
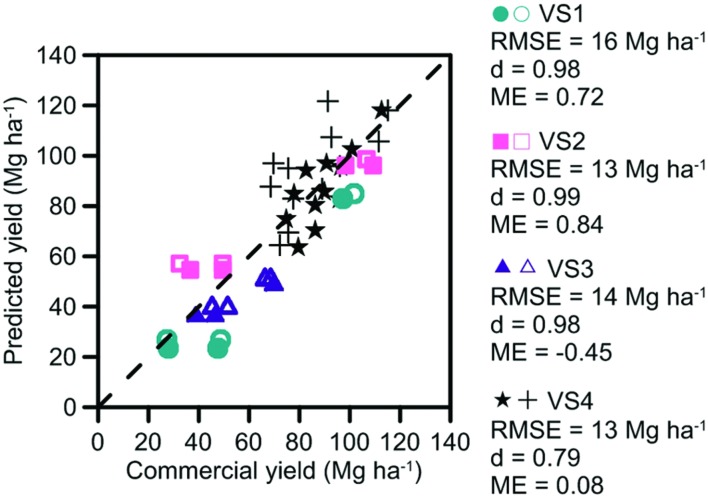
**Measured and predicted commercial yields at the four Validation Sites (VS) used to validate model operation**. Trash was retained on the soil surface as a trash blanket (solid symbols at all VS), incorporated into the soil (open symbols at VS1–3), or burnt after harvest (cross symbols at VS4). RMSE, index of agreement (d) and ME are included.

Sugarcane yields at VS1–4 sites ranged from 27 to 115 Mg ha^–1^ (**Figure 4**) during the experiments. For VS1-3, crops in the first year of the experiment were lodged during a cyclone and produced lower yields (27–52 Mg ha^–1^) than in the second year (66–110 Mg ha^–1^). These differences in yield were well captured by the model, with *d*-values close to a value of one. Both the *d*-value and RMSE measures of model performance indicated that yield was more accurately predicted in the validation (**Figure 4**) than parameterization (**Figure 2**) steps. This may have occurred because the management information used to simulate yields could be obtained from the field experiments at VS1–3 in the validation step, but could only be estimated from mill records for HS and FS in the parameterization step. The larger number of yields measured at VS4 were also well captured by the model with similar RMSE to VS1–3 and a *d*-value of 0.79.

##### SMN

**FIGURE 5:**
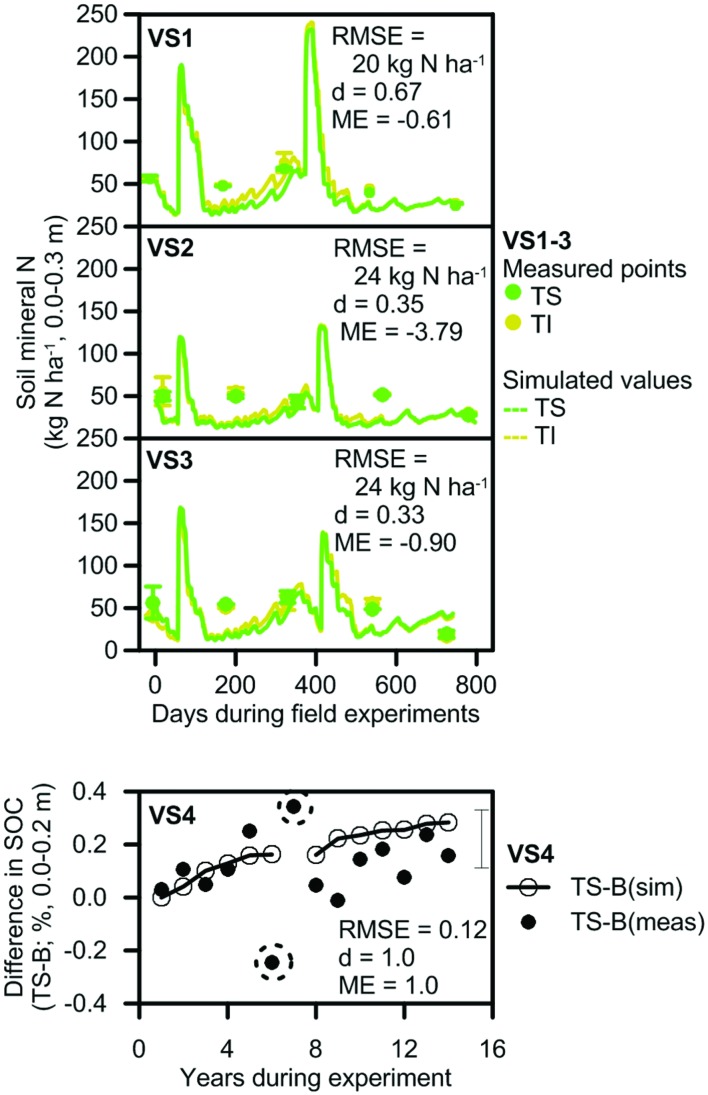
**Measured and simulated values that were used to validate model prediction of SMN at VS1–3 and soil organic carbon (SOC) at VS4**. Measured SMN and SOC are shown as symbols; simulated SMN and SOC is shown as lines. Measurements of SMN include bars that show the range of values. The error bar for SOC shows the standard error of the difference between trash blanketed and burnt values after completion of the experiment. Trash was retained on the soil surface (TS), incorporated into the soil (TI) or burnt (B) after harvest. RMSE, index of agreement (d), and ME are included.

Average SMN at the validation sites was similar between the treatments, but ranged from 14 to 86 kg N ha^–1^ at different measurement times (**Figure 5**). There was a large difference between minimum and maximum SMN measured in the replicates for treatments at the sites, similar to the variability in replicate values that occurred in the small plot data set. On the first, third, and fifth measurement occasions, the largest replicate values were up to 200, 136, and 151% of the smallest measured values on those dates. This contributed to ME values of –0.61, –3.79, and –0.90 at VS1–3, respectively, although the trend in SMN was again captured by the model. Differences between measured and predicted SMN for these sites may have been affected by differences between rainfall received at the sites and at the Babinda weather station. Large (>100 mm) differences in daily rainfall can occur over this distance, which could affect the simulation of nitrate leaching and denitrification and thus lead to differences between measured and predicted SMN.

##### SOC

At VS4, SOC in the trash blanketed treatment increased through time relative to that in the burnt treatment, becoming 0.16% higher after 14 years (**Figure 5**). This increasing trend was generally captured in the simulations. Exceptions were the SOC measurements in years 6 and 7 of the experiment (the circled points for VS4 in **Figure 5**). The difference in SOC between trash blanketed and burnt crops for these measurements was –0.25 and 0.34%, respectively, equivalent to changes in SOC stocks of –5.1 and 7.2 Mg C ha^–1^. While these changes in SOC stocks approximate the total amount of C contained in a trash blanket, they overstate the contribution of trash blanketing to SOC since a substantial proportion (∼40%) of trash C is evolved as CO_2_ during decomposition. Thus these two measurements were regarded as unreliable. When these two points were discarded, measures of model performance were strong with d and ME values of 1.0.

### Model Scenario Results

#### Similarity of Retained and Incorporated Trash Scenarios

There was little difference in yields, N balances or SOC stocks between model scenarios where trash was retained as a trash blanket or incorporated into the soil (data not shown). This was consistent with the field results used in the model parameterization and validation steps in Section “Model Performance,” so the results from model scenarios are presented only for the trash blanket and burnt trash scenarios.

#### SOC

**FIGURE 6:**
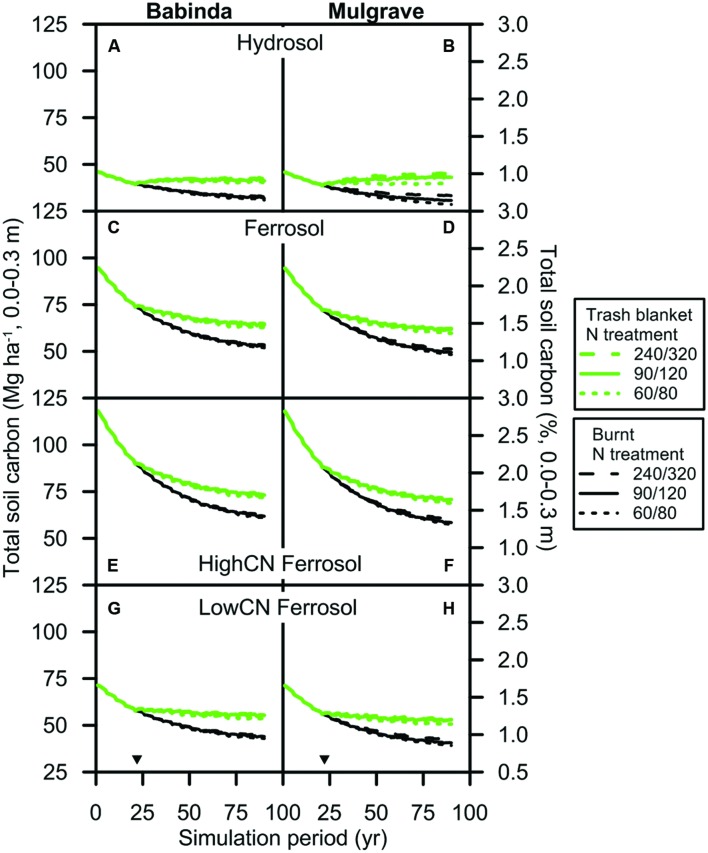
**Soil organic carbon (0.0–0.3 m) in the (A,B) Hydrosol, (C,D) Ferrosol, (E,F) HighCN Ferrosol, and (G,H) LowCN Ferrosol soils subjected to the Babinda and Mulgrave climates**. Trash was managed as a trash blanket (green lines) or burnt (black lines) after harvest. For ease of presentation, three N fertilizer ‘treatments’ that span the N rates simulated are displayed. The codes for these rates are shown in the legend as the combination of N fertilizer rates applied to the plant and ratoon crops: the 60/80 rate (i.e., 60 kg N ha^–1^ applied to the plant crop and 80 kg N ha^–1^ applied to the ratoon crop; dotted lines), the 90/120 rate (solid lines) and the 240/320 rate (dashed lines). Inverted triangles denote start of scenarios with differing trash management and N fertilizer rates following an initial 24-year period with the same management for all scenarios.

Simulated total soil C declined during the first 24 years in all scenarios when trash was burnt (**Figure 6**). Trash × N fertilizer scenarios commenced at this time, and over the next 64 years the rate of change in soil C differed most between the trash treatments: trash blanketing reduced or reversed the decline in C through time. N fertilizer generally had a much smaller effect on soil C (e.g., results for the 30/40, 60/80, and 240/320 N rates, **Figure 6**). Total SOC approached equilibrium concentrations toward the end of the simulation period.

The amount of SOC mineralized from the soils increased as the amount of SOC present at the start of the simulation increased (**Figure 6**). Thus, SOC (0.0–0.3 m) in the scenario soils decreased by 14–21 Mg C ha^–1^ in the Hydrosols, 27–32 Mg C ha^–1^ in the LowCN Ferrosols, 42–45 Mg C ha^–1^ in the Ferrosols and 56–59 Mg C ha^–1^ in the HighCN Ferrosols over the simulation period when trash was burnt (90/120 N rate; Babinda and Mulgrave climates). The SOC for these soils at the beginning of the simulation period was 46, 71, 95, and 118 Mg C ha^–1^, respectively.

The amount of SOC mineralized during the simulation period was reduced when trash was retained. At the end of the simulation period, SOC was 9–13 Mg C ha^–1^ greater when trash was retained instead of burnt for all climate-soil scenarios subjected to N fertilizer ≥ the 60/80 rate. This represented an increase of between 18 and 42% in SOC from retaining trash. The net change in SOC at the end of the simulation period was a trade-off between additions of carbon in trash and mineralization of SOC. For the Hydrosol soils (which had low SOC), there was an absolute increase in SOC (0.0–0.3 m) over the simulation period, e.g., ∼1.3 Mg ha^–1^ (**Figure 6A**) and 3.8 Mg ha^–1^ (**Figure 6B**) when trash was retained (e.g., at the 90/120 N rate). However, for the other soils there was a relative increase in SOC where trash was retained, but a net decrease in SOC over the simulation period in both burnt and trash blanketed systems.

#### Soil N

Total soil N (0.0–0.3 m) changed in a similar pattern to that of SOC in **Figure 6** in response to trash and N scenarios (data not shown). For all climate-soil-N rate combinations, changes in soil N for trash blanketed crops had virtually ceased during the last four crop cycles with net mineralization between 0 and 9 kg N ha^–1^ crop^–1^ during this period. For climate-soil-N rate combinations with burnt trash, total N decreased at a slightly greater rate of 3–13 kg N ha^–1^ crop^–1^ during this period. However, when trash was retained rather than burnt, total N at the end of the simulation period was 702–831 kg N ha^–1^ greater in the Ferrosol soils and 975–1457 kg N ha^–1^ greater in the Hydrosol soils.

#### Crop Yields as Soils Approached Equilibrium Concentrations of C and N

**FIGURE 7:**
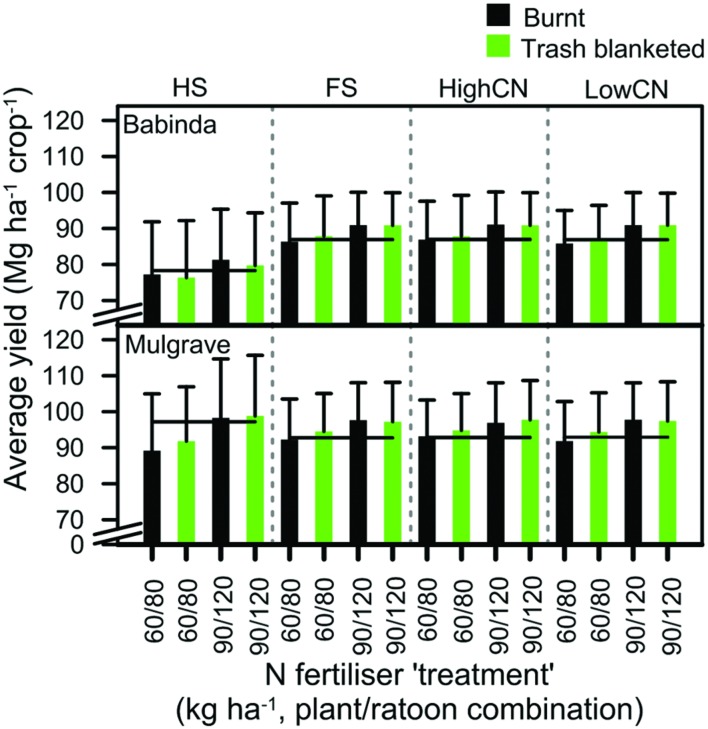
**Mean yield of burnt and trash blanketed crops in the last four crop cycles fertilized with two N fertilizer ‘treatments.’** The codes for these N fertilizer rates are shown in the legend as the combination of N fertilizer rates applied to the plant and ratoon crops: the 60/80 rate (i.e., 60 kg N ha^–1^ applied to the plant crop and 80 kg N ha^–1^ applied to the ratoon crop) and the 90/120 rate. For each location-soil combination, the value for 95% of maximum average yield is shown with a horizontal line. Error bars represent the standard deviation of the mean.

The average yield of crops across all N rates from the last four crop cycles in simulated scenarios ranged from 57 to 93 Mg ha^–1^ for the Babinda climate, and 62 to 103 Mg ha^–1^ for the Mulgrave climate (**Figure 7**). Differences between climates at the two locations tended to have a stronger effect on yield than trash management or N rates equal to or greater than the 60/80 rate. For example, for the Babinda-Ferrosol soil at the 60/80 N rate, yield was 6–7 Mg ha^–1^ higher with the drier Mulgrave climate than the Babinda climate. By comparison, yields for this soil increased by only 2 Mg ha^–1^ if trash was retained instead of burnt, and increased by only 3–5 Mg ha^–1^ if the N rate was increased from 60/80 to 90/120.

**FIGURE 8:**
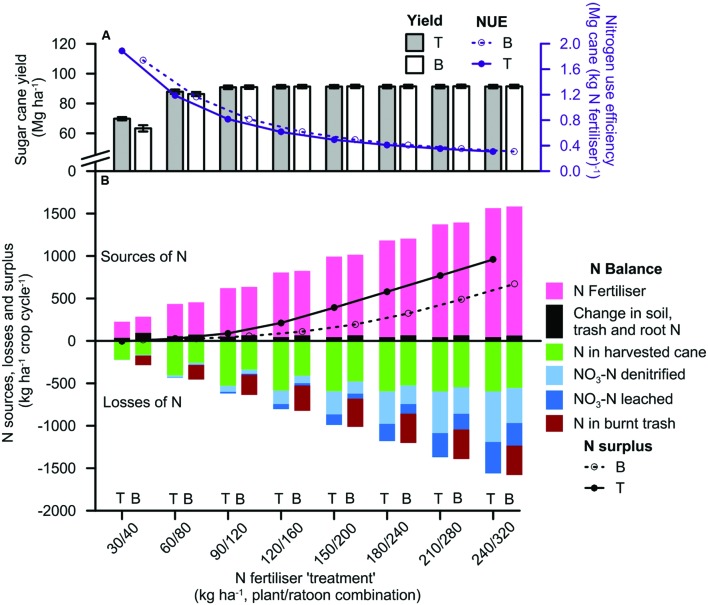
**Simulated (A) cane yield with nitrogen use efficiency (NUE), and (B) N balances with N surpluses, in the management scenarios on the Ferrosol soil with the Babinda climate**. The N fertilizer treatment codes on the horizontal axis represent the combination of N fertilizer rates applied to the plant and ratoon crops (e.g., the 60/80 rate refers to the N fertilizer ‘treatment’ with 60 kg N ha^–1^ applied to the plant crop and 80 kg N ha^–1^ applied to the ratoon crop). Trash was managed as a trash blanket (T) or burnt after harvest (B). Yields are presented as average values ±1 standard error. Positive values in N balances represent inputs of N to the system; negative values represent outputs. Data represent average values from the last four crop cycles. The effect of management scenarios on the yield and N balances in all location-soil combinations was essentially the same, so the results are presented only for the Babinda-Ferrosol scenario.

All crops attained ≥of 95% of maximum yields at the 60/80 or 90/120 rate (**Figure 7**). At N rates greater than the 90/120 N rate, increases in yield were small (≤4 Mg ha^–1^; e.g., the Babinda-Ferrosol in **Figure 8A**). This limited response of yield to increasing rates of N fertilizer led to a corresponding decline in NUE values as N rates increased.

While 95% of maximum yield was achieved with N fertilizer inputs at the 60/80 or 90/120 rate (**Figure 7**), fertilizer was not the only source of N for the scenarios. Net mineralization of soil N provided additional inputs of 2–13 kg N ha^–1^ crop^–1^ to the different climate-soil scenarios (e.g., the Babinda-Ferrosol in **Figure 8B**). These amounts were smaller (2–4 kg N ha^–1^ crop^–1^) for soils that were closer to equilibrium C and N values, such as the Hydrosol and LowCN Ferrosol scenario soils subjected to trash blanketing under either climate (**Figure 6**). However, for soils in which equilibrium C and N concentrations had not been attained, such as the Mulgrave-HighCN Ferrosol scenario soil with burnt trash, the net N mineralized comprised 14% of N inputs to the scenario.

The yield of trash blanketed crops were significantly (*P* < 0.05) greater than the yield of burnt crops only at the 30/40 N rate (e.g., the Babinda-Ferrosol in **Figure 8A**). The yield of burnt and trash blanketed crops at the next highest N rate (60/80) differed by <4 Mg ha^–1^ (**Figures 7 and 8A**). There was a trend for crops at these higher rates to attain 95% of maximum yield at a lower N fertilizer rate than the burnt crop (**Figure 7**), but these N rates occurred within consecutive N fertilizer increments. Consequently, the potential saving in N fertilizer per crop when trash was retained was less than the amount of 40 kg N ha^–1^ crop^–1^ that was applied in consecutive increments (possibly 20 kg N ha^–1^ crop^–1^).

#### Crop Yields during Early Years of Trash Blanketing

**FIGURE 9:**
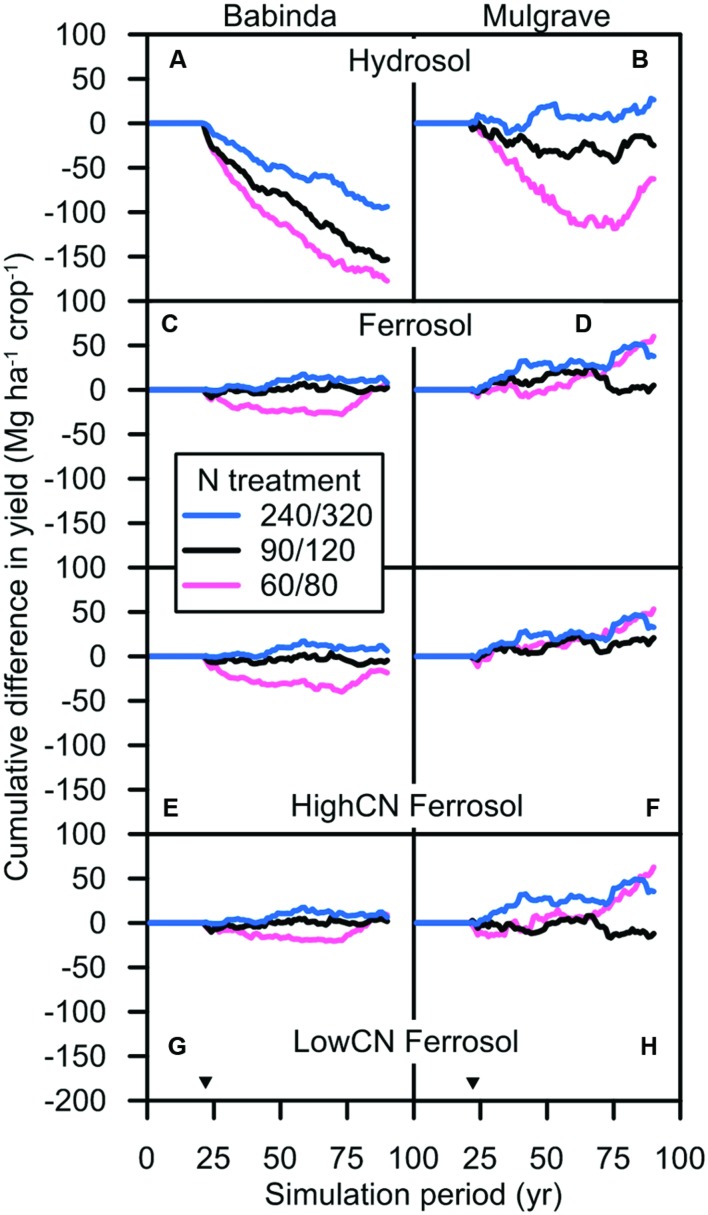
**Cumulative difference between the yield of trash blanketed crops less the yield of burnt crops in different N fertilizer ‘treatments’ in the (A,B) Hydrosol, (C,D) Ferrosol, (E,F) HighCN Ferrosol, and (G,H) LowCN Ferrosol soils subjected to the Babinda and Mulgrave climates**. The N fertilizer treatment codes in the legend refer to the combination of N fertilizer rates applied to the plant and ratoon crops (e.g., the 60/80 rate refers to the N fertilizer ‘treatment’ with 60 kg N ha^–1^ applied to the plant crop and 80 kg N ha^–1^ applied to the ratoon crop). For ease of presentation, three N fertilizer scenarios that span the N rates simulated are displayed: 60/80 (dotted lines), 90/120 (solid lines), and 240/320 (dashed lines). Inverted triangles denote start of scenarios with differing trash management and N fertilizer rates following an initial 24-year period with the same management for all scenarios.

Although trash blanketing had little effect on yield over the last four crop cycles (**Figure 8**) as total soil N approached equilibrium, it reduced yield in at least the early crop cycles after trash blanketing commenced due to immobilization of SMN in the trash blanket (**Figure 9**). The average yield of trash blanketed crops was consistently less than that of burnt crops on the Ferrosol soils by 1–6 Mg ha^–1^ crop^–1^ with, for example, the 60/80 N rate after scenarios commenced. Differences between the yield of trash blanketed and burnt crops continued to occur in some years after this, but the differences were not as large.

On the Hydrosol soils where soil organic N increased in response to trash blanketing (data not shown), trash blanketing suppressed yield for a longer period. For these crops, yields may have been limited by waterlogging (see section APSIM-Sugar) in addition to immobilization of N in trash. At the Babinda-Hydrosol, the yield of trash blanketed crops was consistently less than that of burnt crops by 1–11 Mg ha^–1^ for the first 20 years after scenarios were implemented. After this time, the average difference between trash blanketed and burnt yields was reduced to ≤2 Mg ha^–1^ after 30 years at N rates ≤ the 90/120 rate, or after 12 years at higher N rates. At the Mulgrave-Hydrosol, trash blanketed crop yields were consistently less than burnt crop yields by up to 4 Mg ha^–1^ for the first 4 years. For this climate-soil combination, the yield difference between trash blanketed and burnt crops was reduced to ≤2 Mg ha^–1^ after 18 years at all N rates.

#### N Balances

Average N balances were determined for the final four crop cycles for both trash blanketed and burnt trash systems at all N rates in the scenarios. The relationship between yield and input and output terms were consistent across all location-soil scenarios, so the results have been described by reference to the Babinda-Ferrosol scenario (**Figure 8B**).

##### Input terms

Fertilizer was the main input term in the balances at all N rates, but changes in soil organic N were also an input (**Figure 8B**). N surpluses (difference between N inputs and N removed in harvested cane and burnt trash) were greater for trash blanketed than burnt scenarios, but in both cases were less than 15 kg N ha^–1^ year^–1^ at N rates ≤ 90/120 rate.

##### Output terms

Nitrogen removed from the system in harvested cane increased in both burnt and trash blanketed systems as the N rate increased from the 30/40 to the 150/200 rate (**Figure 8B**). Given that yields were little affected by N rate (with the exception of the lowest N rate, **Figure 8A**), the higher N in cane was mainly caused by stalk N concentrations increasing with higher N inputs. For example, cane N concentrations increased from 0.17 to 0.35% in burnt crops and 0.21 to 0.43% in trash blanketed crops over the range of N fertilizer rates. At N rates above the 150/200 rate, the N removed in cane in the trash blanketed system was virtually constant (**Figure 8B**), and reached a maximum value of 595 kg N ha^–1^ crop cycle^–1^. In the burnt system, N in harvested cane increased slightly with increasing N input above the 150/200 rate N to a maximum of 554 kg N ha^–1^ crop cycle^–1^ at the 240/320 N rate.

Nitrogen lost to the environment from burning trash was substantial in burnt trash scenarios (e.g., the Babinda-Ferrosol, **Figure 8B**) and was the main cause of the lower N surplus for this scenario. The amount of N removed by burning trash was related to yield and therefore was relatively insensitive to N inputs ≥ the 90/120 N rate (**Figure 8A**). The amount of N lost in combined nitrate leaching and denitrification was small at N rates ≤ the 90/120 rate (e.g., average 12 kg NO_3_-N crop^–1^ for the Babinda-Ferrosol, **Figure 8B**). However, these losses increased at an increasing rate (e.g., average 160 kg NO_3_-N crop^–1^ for the Babinda-Ferrosol).

Differences between the trash retained and burnt trash scenarios in the N removed in harvested crops and from burning trash led to lower total environmental losses from trash blanketed scenarios (**Figure 8B**), especially below the 150/200 rate. Thus, reducing N fertilizer from the 90/120 to the 60/80 N rate approximately halved environmental losses from the trash blanketed system but had a smaller effect on losses in the burnt system. This result was consistent across all climate-soil combinations.

## Discussion

Green cane trash blanketing had little effect on commercial crop yields relative to burnt crop systems (**Figures 7** and **8**). While total soil N and stalk N was greater for trash blanketed than burnt crops, this did not translate into greater yields when the systems were compared at fertilizer rates at or above the 90/120 rate. Crop yields were essentially the same at this and greater N rates in both burnt and trash blanketed systems, indicating that the potential to increase yield was limited by factors other than N. The yield of crops that were fertilized with the same rate of N fertilizer improved markedly from the Babinda to the Mulgrave climates (**Figure 7**). Since the differences between the locations were predominantly due to rainfall (**Figure 1**), then it appears that trash blanketed crops at Babinda could not respond to the additional N supplied from retained trash due to the rainfall-induced limitations such as waterlogging and crop lodging. This observation is consistent with greater yields recorded in drier years for the calibration data set (see Parameterization section). The limiting effect of high rainfall on crop yields was pronounced for the Babinda-Hydrosol (**Figures 7A and 9A**). This soil is characterized by shallow water tables (Isbell, 2002), and when combined with the wetter Babinda climate, resulted in lower yields for trash blanketed than burnt crops (**Figure 7**). Accordingly, simulated crops attained ≥95% of maximum yields for all location-soil combinations at a N rate of 90/120 regardless of trash management.

For well-drained soils subject to drier climates (e.g., < ∼2,000 mm year^–1^), trash blanketing has been found to reduce soil water evaporation and thereby improve yield by both retaining more water in the soil in addition to providing a source of N (Wood, 1991; Thorburn et al., 2002). These observations compare with crops grown on the Ferrosol soils at Mulgrave, where the average difference between trash blanketed and burnt crop yields was small (**Figure 7**), but greater than at Babinda and consistent with field measurements (Wood, 1991).

While the maximum yields obtained by the trash blanketed and burnt crops were essentially the same for any location-soil combination, it can be seen that average yields ≥95% of maximum values are achieved in crops from trash blanketed scenarios at lower N rates than those in burnt trash scenarios (an exception being the Babinda Hydrosol discussed above; **Figures 7 and 8A**). The saving in N is a smaller contribution of N than the 40 kg N ha^–1^ crop^–1^ increment between N fertilizer scenarios in our simulations, e.g., possibly in the order of 20 kg N ha^–1^ crop^–1^. While a reduction of this magnitude may seem insignificant at the field or farm scale, it is more meaningful when aggregated to a regional level. For example, a reduction of this order would equate to 2.7 Mg of N over the 1,364 km^2^ of the Australian Wet Tropics sugarcane area (Australian Government, 2013). Such a reduction is important given the need to reduce N losses from catchments in this region (Kroon et al., 2016). Reduced N fertilizer applications would also reduce N_2_O emissions from sugarcane farms (Thorburn et al., 2010).

A reduction in the N fertilizer rates for trash blanketed crops during the first crop cycles after trash blanketing was introduced led to a decrease in simulated crop yields for 6–30 years (**Figure 9**). This occurred because the trash blanketed crops were subjected to N stress due to the immobilization of N in the added trash. Maintaining N fertilizer at the level applied to burnt crops provided N to compensate for this immobilization demand. The duration of this phase of increased N immobilization coincides with the period that trash blanketing has been practiced on many Australian farms in the wet tropics region. However, in later crop cycles as soil N approaches equilibrium and the immobilization demand was met by increased N mineralizing capacity of the trash-blanketed soil, surplus N was prone to loss. Substantial SMN measured throughout the year in trash blanketed soils of the Queensland wet tropics region, e.g., 20–70 kg N ha^–1^ in surface (0.0–0.3 m) soils and 80–160 kg N ha^–1^ over greater depths (0.0–1.5 m; Thorburn and Goodson, 2005; Meier et al., 2006b), and low recovery of fertilizer by crops (Meier et al., 2006b), suggests that crops may be well supplied with N. However, the limited capacity for crops to utilize the additional N supplied from trash blanketing may be related to the relationship between N supply during trash decomposition and crop demand. Trash blankets decompose throughout the year following deposition at harvest (Robertson and Thorburn, 2007a). The period of ‘grand growth’ for sugarcane typically occurs between 150 and 280 days after harvest (Fageria et al., 2010), which may not coincide with the availability of N from trash. For example, around 20% of the N in decomposing trash blanket was found to be released during this period but this represented only 5–10 kg N ha^–1^.

A total of 9–12 Mg C ha^–1^ was stored in trash blanketed soils relative to burnt soils as soil C and N approached equilibrium concentrations at the end of the simulation period (**Figure 6**). Total SOC is the product of SOC decomposition and accumulation processes that, in response to trash blanketing, led to a relative or absolute increase in SOC depending on initial SOC concentrations. Thus, there was an overall increase in SOC for the Hydrosol soils with low initial SOC (<1% in the surface 0.3 m soil layer) when trash was retained (**Figures 6A,B**). In the Ferrosol soils (**Figures 6C–H**), the amount of SOC decomposed exceeded the amount accumulated in both trash management systems. However, SOC declined less under trash blanketing and so there was a net avoidance of CO_2_ emissions when trash was retained instead of burnt. The simulated potential for long term trash blanketing to increase SOC stocks over time by modest amounts in surface soil layers (≤0.25 m) is generally consistent with field data (Blair et al., 1998; Robertson and Thorburn, 2007b; Thorburn et al., 2012). Measured increases in SOC do not necessarily occur in proportion to the period of time that trash blanketing has been practiced and changes at greater depths are variable. These differences have been attributed in some cases to dilution of SOC throughout the profile due to cultivation (Blair et al., 1998; Thorburn et al., 2012), which was not included in this analysis.

Despite these past increases in SOC stocks in response to trash blanketing, the potential to further increase SOC by trash blanketing is likely to be limited for the North Queensland wet tropics region. Trash blanketing is now practiced on virtually all sugarcane farms in this region, with uptake commencing several decades ago (Wood, 1991; Robertson and Thorburn, 2007a). Thus, SOC stocks could be approaching equilibrium values (**Figure 6**) and much of the gains in SOC may already have been achieved. As a consequence, the adoption of reduced N fertilizer rates in response to trash blanketing represents an important ongoing means of mitigating climate change for the sugar industry in this region.

## Conclusion

This study indicated that N inputs from trash blanketing in wet tropical sugarcane systems could provide a modest contribution to the crop’s N requirement and thus permit the rate of N fertilizer to crops to be reduced. This practice could reduce environmental losses of N and contribute to GHG mitigation by reducing N_2_O emissions and increasing SOC stocks. While potential savings in N fertilizer use were modest at the field scale, they have potential to be important when aggregated at the regional level. Incorporation of trash in this environment had little effect on crop yield or SOC stocks compared to when trash was retained on the soil surface as a trash blanket.

## Author Contributions

EM: Conducted model simulations, analyzed model output, prepared manuscript including figures and tables. PT: Provided supervision and advice on overall project, provided extensive suggestions and revisions to manuscript.

## Conflict of Interest Statement

The authors declare that the research was conducted in the absence of any commercial or financial relationships that could be construed as a potential conflict of interest.
