# Supplementary material for: Long Term Sugarcane Crop Residue Retention Offers Limited Potential to Reduce Nitrogen Fertilizer Rates in Australian Wet Tropical Environments
Source: Front Plant Sci. 2016 Jul 12;7:1017. doi: 10.3389/fpls.2016.01017 (PMC4940410; doi:10.3389/fpls.2016.01017)
Supplement: Supplementary file 1 [file Table_1.PDF]

Supplementary Material

APSIM soil module parameters

Elizabeth A. Meier*, Peter J. Thorburn

*** Correspondence:** Corresponding Author: Elizabeth.Meier@csiro.au

1 Supplementary Data

Supplementary Table 1. APSIM soil module parameters for the Hydrosol Site (HS), Ferrosol Site (FS) and the four Validation Sites (VS)

(a) Single value parameters at sites

Parameter	Sites					
	HS	FS	VS1	VS2	VS3	VS4
Coefficient second stage evaporative demand	3.0	3.0	3.0	5.0	3.0	3.0
Soil albedo	0.28	0.11	0.25	0.25	0.22	0.1
Soil C:N ratio	9.6	16.7	9.6	11.3	10.5	10.5
Fraction of plant available water capacity initially available	0.60	0.71	0.90	1.15	1.14	0.8

(b) Parameters values by soil layer

Site	Parameter	Soil layer (0.0 to 1.5 m depth)						
		1	2	3	4	5	6	7
All	Depth (m)	0.15	0.15	0.15	0.15	0.30	0.30	0.30
HS	pH	5.1	4.9	4.9	4.9	4.9	4.9	4.9

Site	Parameter	Soil layer (0.0 to 1.5 m depth)						
		1	2	3	4	5	6	7
	Inert fraction of initial SOC	0.30	0.35	0.55	0.90	0.95	0.95	0.99
	Soil water (-1,500 kPa; mm mm^{-1})	0.22	0.23	0.21	0.20	0.20	0.17	0.16
	Soil water (-33 kPa; mm mm^{-1})	0.34	0.35	0.33	0.33	0.32	0.30	0.30
	Soil water (-1 kPa; mm mm^{-1})	0.38	0.38	0.50	0.50	0.46	0.47	0.42
	Bulk density (Mg m^{-3})	1.63	1.63	1.32	1.32	1.44	1.41	1.53
	Profile drainage rate coefficient	0.3	0.3	0.3	0.3	0.1	0.1	0.1
	SOC (%)	1.13	0.83	0.68	0.55	0.40	0.24	0.14
	fbiom[1]	0.12	0.10	0.05	0.01	0.01	0.01	0.01
	NH_4^+-N (ppm)	5.67	4.31	3.27	2.45	1.33	1.04	1.06
	NO_3^--N (ppm)	0.04	0.02	0.00	0.00	0.00	0.00	0.00
FS	pH	4.9	4.9	4.9	4.9	4.9	4.9	4.9
	Inert fraction of initial SOC	0.30	0.35	0.55	0.90	0.95	0.95	0.99
	Soil water (-1,500 kPa; mm mm^{-1})	0.30	0.33	0.31	0.32	0.34	0.32	0.38
	Soil water (-33 kPa; mm mm^{-1})	0.38	0.39	0.39	0.39	0.39	0.38	0.40
	Soil water (-1 kPa; mm mm^{-1})	0.47	0.46	0.49	0.49	0.46	0.48	0.42
	Bulk density (Mg m^{-3})	1.40	1.44	1.36	1.36	1.44	1.38	1.54
	Profile drainage rate coefficient	0.30	0.30	0.30	0.25	0.25	0.25	0.25
	SOC (%)	2.54	2.11	1.47	1.07	0.97	0.81	0.45

Site	Parameter	Soil layer (0.0 to 1.5 m depth)						
		1	2	3	4	5	6	7
	fbiom[1]	0.08	0.07	0.04	0.01	0.01	0.01	0.01
	NH_4^+-N (ppm)	4.13	3.07	2.56	1.98	1.71	3.36	1.77
	NO_3^--N (ppm)	0.20	0.03	0.03	0.71	3.47	2.61	2.52
VS1	pH	5.1	4.9	4.9	4.9	4.9	4.9	4.9
	Inert fraction of initial SOC	0.35	0.40	0.55	0.90	0.95	0.95	0.99
	Soil water (-1,500 kPa; mm mm^{-1})	0.19	0.20	0.19	0.17	0.14	0.14	0.14
	Soil water (-33 kPa; mm mm^{-1})	0.42	0.42	0.40	0.40	0.39	0.38	0.40
	Soil water (-1 kPa; mm mm^{-1})	0.48	0.47	0.46	0.44	0.45	0.47	0.46
	Bulk density (Mg m^{-3})	1.30	1.33	1.38	1.42	1.40	1.34	1.37
	Profile drainage rate coefficient	0.70	0.70	0.70	0.70	0.70	0.70	0.70
	SOC (%)	1.26	1.02	0.69	0.59	0.40	0.30	0.23
	fbiom[1]	0.12	0.10	0.05	0.01	0.01	0.01	0.01
	NH_4^+-N (ppm)	2.96	4.36	6.80	5.60	5.00	2.47	1.70
	NO_3^--N (ppm)	11.22	9.51	4.08	2.44	1.50	0.76	0.64
VS2	pH	5.1	4.9	4.9	4.9	4.9	4.9	4.9
	Inert fraction of initial SOC	0.35	0.40	0.55	0.90	0.95	0.95	0.99
	Soil water (-1,500 kPa; mm mm^{-1})	0.15	0.15	0.18	0.18	0.17	0.16	0.16
	Soil water (-33 kPa; mm mm^{-1})	0.32	0.30	0.38	0.36	0.36	0.36	0.35

Site	Parameter	Soil layer (0.0 to 1.5 m depth)						
		1	2	3	4	5	6	7
	Soil water (-1 kPa; mm mm^{-1})	0.43	0.44	0.40	0.42	0.43	0.40	0.39
	Bulk density (Mg m^{-3})	1.46	1.42	1.53	1.48	1.46	1.55	1.56
	Profile drainage rate coefficient	0.50	0.50	0.50	0.50	0.50	0.50	0.50
	SOC (%)	1.29	1.07	0.78	0.67	0.64	0.57	0.36
	fbiom[1]	0.12	0.10	0.05	0.01	0.01	0.01	0.01
	NH_4^+-N (ppm)	7.58	7.89	5.88	4.67	3.08	3.37	2.10
	NO_3^--N (ppm)	2.78	2.79	1.37	1.15	1.04	0.98	0.92
VS3	pH	5.1	4.9	4.9	4.9	4.9	4.9	4.9
	Inert fraction of initial SOC	0.35	0.40	0.55	0.90	0.95	0.95	0.99
	Soil water (-1,500 kPa; mm mm^{-1})	0.19	0.20	0.24	0.24	0.22	0.19	0.16
	Soil water (-33 kPa; mm mm^{-1})	0.36	0.35	0.36	0.36	0.36	0.36	0.35
	Soil water (-1 kPa; mm mm^{-1})	0.45	0.46	0.41	0.40	0.40	0.39	0.38
	Bulk density (Mg m^{-3})	1.39	1.36	1.51	1.59	1.58	1.57	1.59
	Profile drainage rate coefficient	0.30	0.30	0.30	0.30	0.10	0.10	0.10
	SOC (%)	1.37	1.23	0.72	0.61	0.44	0.30	0.23
	fbiom[1]	0.12	0.10	0.05	0.01	0.01	0.01	0.01
	NH_4^+-N (ppm)	3.70	8.44	4.66	4.24	3.50	2.15	1.32
	NO_3^--N (ppm)	7.91	6.63	4.43	2.29	1.19	0.76	0.74

Site	Parameter	Soil layer (0.0 to 1.5 m depth)						
		1	2	3	4	5	6	7
VS4	pH	5.0	5.0	5.0	5.5	6.0	6.0	6.0
	Inert fraction of initial SOC	0.2	0.4	0.55	0.85	0.95	0.95	0.99
	Soil water (-1,500 kPa; mm mm^{-1})	0.18	0.18	0.18	0.18	0.18	0.16	0.14
	Soil water (-33 kPa; mm mm^{-1})	0.32	0.32	0.32	0.32	0.32	0.30	0.28
	Soil water (-1 kPa; mm mm^{-1})	0.52	0.52	0.50	0.50	0.49	0.49	0.48
	Bulk density (Mg m^{-3})	1.02	1.02	1.07	1.07	1.07	1.07	1.07
	Profile drainage rate coefficient	0.4	0.4	0.4	0.4	0.4	0.4	0.4
	SOC (%)	0.82	0.82	0.68	0.50	0.25	0.20	0.20
	fbiom[1]	0.10	0.07	0.05	0.03	.02	.01	.01
	NH_4^+-N (ppm)	1.0	1.0	1.0	1.0	1.0	0.5	0.5
	NO_3^--N (ppm)	2.0	2.0	2.0	2.0	1.0	1.0	1.0
HS, FS	Root expansion factor (Babinda)	1.0	1.0	0.5	0.1	0.1	0.0	0.0
HS, FS	Root expansion factor (Mulgrave)	1.0	1.0	0.5	0.5	0.5	0.5	0.5
All	Water extraction parameter	0.10	0.10	0.10	0.10	0.08	0.08	0.04

[1]fbiom, fraction of initial non-inert C as microbial biomass
